# Measuring the Changes in Aggregate Cycling Patterns between 2003 and 2012 from a Space Syntax Perspective

**DOI:** 10.3390/bs4030278

**Published:** 2014-08-08

**Authors:** Stephen Law, Fernanda Lima Sakr, Max Martinez

**Affiliations:** 1 Bartlett School of Graduate Studies, University College London, 132 Hampstead Road, London, NW1 2BX, UK; E-Mails: fesakr@gmail.com (F.L.S.); m.martinez@spacesyntax.com (M.M.); 2 Space Syntax Limited, 21 Brownlow Mews, London, WC1N 2LG, UK

**Keywords:** spatial cognition, space syntax, spatial configuration, cyclist movement, cycling route choice

## Abstract

There has been a world-wide surge of interest in cycling over the last 10 years of which London has seen a continuous growth in cyclists and investment in infrastructure that has resulted in the introduction of the Barclays Cycle Superhighway and Barclays Cycling Hiring Scheme. Despite the investment in cycling infrastructure, there has been little understanding of cycling activity patterns in general and the effect of spatial configuration on cycling route choices. This research aims at measuring the impact of cycling infrastructure and spatial configuration on aggregate cyclist movement over two time periods. To do so, this paper presents a spatial-based cyclist movement statistical model that regress cyclist movement flows with measure of spatial configuration, safety and infrastructure and urban character attributes. Using Elephant and Castle, a Central London location, as a case study, the authors analyze cycling movement data sets from 2003 and 2012 to compare the change in cycling behaviour and the impact that the Cycling Superhighway 07, introduced in 2011, has had on cycling patterns. Findings confirm the growth of cycling in London with a 1000% increase in cyclists along some routes in comparison to a 10% increase in population at the same time. More importantly, results also suggest that higher cyclist movement were observed along routes with greater convenience and continuity—over and above route segregation from vehicular traffic. The relationship between spatial configuration and aggregate cyclists movement is consistent between 2003 and 2012 where spatial configuration have remained the same while changes were observed in both modal split and cycling infrastructure. This result is in line with previous research wherein aggregate higher cyclists movement are observed on major routes offering direct connections than less direct routes. From a spatial cognition perspective, this research enriches our understanding on how the external built environment as measured by the spatial configuration measure relates to aggregated cyclists movement overtime and in identifying key potential factors in influencing cyclist wayfinding. Further research is needed into validating the results and examining this relationship at an individual basis on route choice. These results help us better understand the trade off between cycling safety and cycling legibility which could help inform cycling route design in the future.

## 1. Introduction

The year of 2007 marked the transition from a majority of rural world population to one dominated by urban dwellers. Among other things, this shift to cities has increased the need to improve sustainable and active forms of transport. e.g., walking and cycling. With the reassessment of the value of living in central areas a number of cities have managed a reduction in the use of private motor vehicles. Consequently, cycling has moved to the forefront of the design, planning and transport agenda [1]. For example, cycling in London is growing at a fast pace, with reported increase over the last decade by 70% and on major roads by 173%, as recorded by Transport for London (TfL) on the Transport for London Road Network (TLRN) [1,2]. London also aims to achieve a target growth of a 400% increase in cycling by 2026 compared with 2001 levels (107.2% increases recorded in 2008 over 2001 base levels). Along the uptake of cycling, London’s mayor has announced a 1 billion pound budget for cycling projects in London. The Barclays Cycling Hiring Scheme popularly referred to as “Boris bikes” continues to grow as does the London Cycle Superhighway network.

Despite continuous investment in cycling infrastructure, there is a lack of an evidence-based understanding of cycling activity patterns and in particular on the role that of spatial configuration has on cycling activity. The focus has largely been on developing design guidance for the implementation of cycling infrastructure and a focus on the typology of this infrastructure: advanced stop lines, segregated lanes, cycle tracks, cycle crossings. Another area that has received considerable attention, particularly in the US is the distinction between segregated cycle tracks or “vehicular cycling” were cycle routes share the road with other vehicles. However, there have been very few studies that investigate the resulting route choice effect of all the local infrastructure implementations.

The surge of interest in cycling represents an opportunity to study the attributes influencing cycling route choice and more interestingly to look at the evolution of cycling movement patterns. The aim of this paper is therefore to quantify the shift in cycling activity and to test what impact the implementation of cycling infrastructure has had on these. These results can help us better understand the trade off between cycling safety and cycling legibility which can help inform cycling route design in the future.

## 2. Research Question

In transportation, cycling research has focused mostly on stated preference studies. Previous transport stated preference studies have identified the following criteria influencing cyclist movement: distance, time, effort, number of junctions, number of traffic lights, pleasantness, attractions, quality of pavement, protection from weather, crowdedness, gradient, and personal and traffic safety [3]. In a study to identify the extent of these criteria; distance, pleasantness and safety were identified as the most important attributes for cyclists [4]. While stated preference study can improve validation of cycling route choice, the results must still be verified by actual behavior. Recent revealed preference study began to fill this gap. A revealed preference study conducted in Zurich identified trip length as the most important factor in influencing cycling route choice [5]. Another revealed preference study, conducted in San Francisco, identified bicycle lanes, number of turns, distances, and the slope as the important attribute in influencing cycling route choice whilst traffic volume, traffic speed, number of lanes and crime rates and nightfall had no significant effects [6]. What appears missing in transportation research are threefold; first, from a spatial cognition perspective the need to better understand both the internal processes and external environments influences on wayfinding, second, a better understanding on the tradeoff between legibility and safety and third, on how cycling activity patterns changes overtime. All of which can provide useful insights on future cycling trends and in designing future cycling infrastructure.

This paper builds on previous research using the techniques and methods of space syntax to quantify the built environment in relation to cyclists movement activity overtime. Space syntax applies methods in graph theory to study the configuration of spatial networks in cities built environment [7]. In spatial cognition research, Peponis studied the relationship between configuration properties and observed pedestrian behaviour [8]. Following this seminal study, different aspect of wayfinding have been successfully examined using space syntax analysis including wayfinding in virtual environments [9] complex buildings such as and multi-level buildings [10], as well as relating space syntax measures to resident’s mental map [11]. Recent methodological development expands on the theory in stating that angular distance [12] in measuring accessibility provides a more accurate representation of aggregate pedestrian movement distribution than metric distance and topological distance. In transport studies, space syntax measures were shown empirically to relate strongly to pedestrian flows, vehicular flows [12,13] and public transport passenger volume [14]. In cycling research, Radford, Gil, and Chiaradia found significant correlations between spatial configuration and observed aggregate cycling movement through a regression framework [15]. The research also found inconclusively that cyclist at an individual level follows neither metric shortest path nor angular shortest path. What appears missing in previous space syntax literature on cycling behaviour is twofold; one is the limited research in cycling route choice and wayfinding at an individual basis and secondly limited research looking at how cycling movement pattern changes overtime.

This paper aims to extend previous cycling research on the latter by examining changes in aggregate cycling movement pattern overtime and specifically studying the relationship between cycling safety and cycling accessibility. 

## 3. Research Approach

This research uses a cyclist movement patterns sample collected in London’s Elephant and Castle area in 2003 and 2012. During this period the area has seen minor changes in spatial configuration but has experienced changes in both transport modal split and the provision of cycling infrastructure. This allows the study of interactions between the changes in overall cycling volume and the changes in cycling infrastructure within a constant spatial configuration framework.

1. We first look at how cyclist movement patterns have changed overtime in the case study area that has experienced a significant transport modal shift in the last 10 years, and link the analysis of cycling movement and its changes through time with changes cycling infrastructure within a relative stable spatial configuration.
2. We then proposed a cyclist movement model where aggregate cyclists movement is correlated to spatial configuration measures and cyclists infrastructure in 2012.

### 3.1. Cyclists Movement Analysis

**Figure 1:**
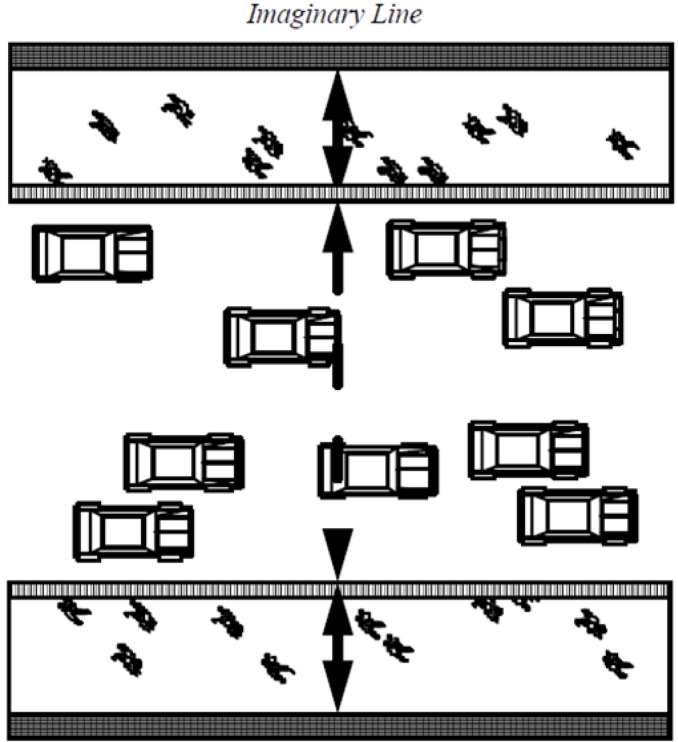
Gate count method.

This section describes in detail the first stage of the research where cyclist movement patterns are collected, described, explored and compared between the two time periods. Gate count method is applied to record the movement patterns of bicycles. In the first step, gates are selected on all cyclist accessible space within the study area. In the second step, an imaginary line is drawn across each gate where cyclists are counted whenever this imaginary line is crossed. Counts are recorded for twelve hours per day, which are then aggregated into an average cyclists per hour formats. More details are illustrated in the case study section. Figure 1, below, illustrates the imaginary line in the gate count method. This observation method is repeated for the two case study years, 2003 and 2012 where the data will be analysed statistically and visually through mappings.

### 3.2. Cyclist Movement Model

This section describes in detail the second stage of the research where aggregate cyclist movement is correlated with three criteria named accessibility and transport, safety and infrastructure and character and land use. Data for each criteria were collected on each route and gate. The method of ordinary least square (OLS) is used for the estimation of the Normal-Linear-Quadratic model (NLQ model,) where Log Cyclists movement of each gate is regressed against a set of Accessibility, Safety and Character parameters (1).

*Log*(*Cyclists movement*) = ∑_i_*α*_i_*Accessibility* + ∑_k_*β*_i_*Safety* + ∑_k_γ_i_*Character* + *ε* (1)

Variables for each criteria are explored in the case study through a stepwise regression method in testing its statistical significance and selecting the most statistically significant model. The stepwise regression method offers the flexibility to incorporate different levels and both qualitative and quantitate evidence for comparison. This would be further validated by constructing four separate regression models and comparing the results within each. Future research will improve on the specification of the cyclist movement model, sample size and to provide more detail instruments in measuring safety, character, route pleasantness and level of infrastructure intervention.

**Figure 2:**
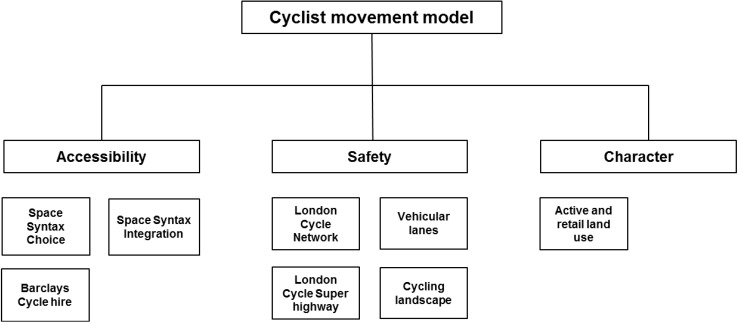
Proposed Cyclist movement model.

Figure 2 and Table 1 summarise the variables to be tested in the cyclist movement model. The following section will describe in detail the specification of each variable.

**Table 1:** Cyclist movement model variables.

Type	Variables	Source
Accessibility and transport	Space Syntax NA Choice	Space Syntax Limited
	Space Syntax NA Integration	Space Syntax Limited
Safety and infrastructure	London Cycle Network	TfL
	London Cycle Superhighway	TfL
	London Cyclist landscape	Site visits/Google Streetview
	Number of vehicular lanes	Site visits/Google Streetview
Character and Land use	Active land use	Site visits/Google Streetview

### 3.3. Accessibility

Accessibility had previously been identified as key criteria in influencing aggregate cyclists movement [15]. This study proposes two spatial configuration measures of accessibility for the cyclist movement model; space syntax angular integration and space syntax normalized angular choice.

#### 3.3.1. Space Syntax Angular Integration

In a space syntax network model, the interrelationship between each segment of a street network is analysed. One measure is Space Syntax Angular Integration (2) also known as closeness centrality in graph theory measures the reciprocal of the sum of the shortest paths between every origin to every destination [7,12,16].

*C_c_*(*P*_i_) = 1/(∑_k_*d*_ik_) (2)

The space syntax angular integration variable is specified as a continuous variable where the following radiuses; 1200 metres, 2000 metres, 3000 metres, 5000 metres, and N are calculated for each segment and for each observation gate.

#### 3.3.2. Space Syntax Normalised Angular Choice

A second measure is Space Syntax Normalised Angular Choice (3) which measures the through movement or betweenness of a route [17]. This measure has been found to correlate highly to different scales of movement.


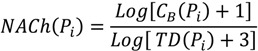
(3)

The measure is made up of two components; Angular choice/betweenness (4) which measures how many shortest paths overlap between all pairs of origins and destinations [12,17,18] and Total Angular Depth (5), which measures the sum of the shortest paths between every origin to every destination.


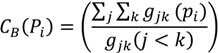
(4)

*TD*(*P*_i_) = (∑_k_*d*_ik_)^1^ (5)

The space syntax normalised angular choice variable is specified as a continuous variable where the following radiuses; 1200 metres, 2000 metres, 3000 metres, 5000 metres, and N are calculated for each segment and for each observation gate.

### 3.4. Safety and Infrastructure

Safety and infrastructure had previously been identified as key criteria in influencing cycling route choice [3,4]. Four measures of safety and infrastructure have been proposed for the cyclist movement model. The propose variables include, the presence of the London Cycle Network, the presence of London Cycle Superhighway, the presence of London cycling landscape, and the number of vehicular lanes. More research is needed in identifying accurate instruments in measuring safety and infrastructure. One possibility is to define a cycling landscape quality index indicating differing levels of interventions and perceived safety.

#### 3.4.1. London Cycle Superhighway

**Figure 3:**
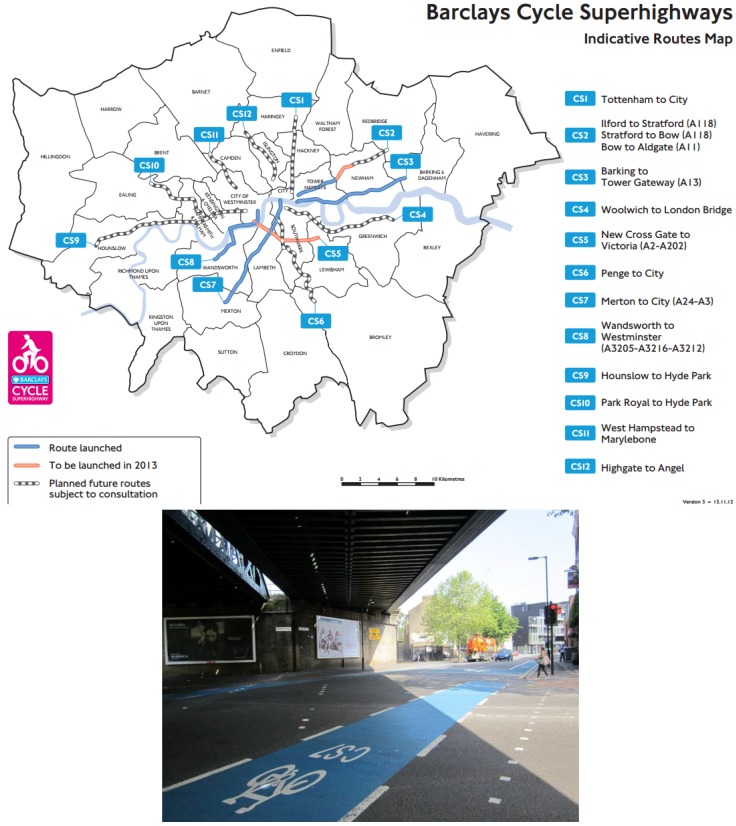
The map highlights the location of various London Cycle Superhighways and an image of its landscape design [19,20].

The first measure of safety and infrastructure is the London Cycle Superhighway. The London Cycle Superhighway was announced by London Major Ken Livingstone in 2008 with the aim of creating continuous cycling routes into central London [19]. Cycle Superhighways (CS) are designed to be direct, continuous, comfortable, easy to find and safe. There are a total of 12 routes planned and as of 2014, only four routes are in use; this includes CS8 from Wandsworth to Westminster, CS3 from Barking to Tower Gateway, CS7 from Merton to the City and CS2 from Stratford to Aldgate. CS 5 is currently under consultation. Safety, priority and junction design are important consideration in the design of the Superhighways. Figure 3 illustrates all the London Cycle Superhighway in the city and the photo describes the dedicated visible blue lanes for cyclist. This variable is specified as a dummy variable where 0 indicates the gate is not on London Cycle Superhighway and 1 indicates the gate is located on London Cycle Superhighway.

#### 3.4.2. Cycling Landscape

The second measure of safety and infrastructure is the cycling landscape infrastructure provision. This dataset has been observed directly on site and verified digitally on Google Streetview. Routes with provision of cycling landscape are separated by its levels of intervention. This includes routes that are marked with a cycling sign on the surface, dedicated cycle lane that is part of the street network and dedicated cycle lane that is segregated from the street network [21]. Two variables are identified for the cyclist movement model, cyclist marked routes and cyclist landscape. Cyclist marked routes is defined as a dummy variable where 0 indicates the gate is on a route that is not marked with a cycling sign on the surface and 1 indicates the gate is on a route that is marked with a cycling sign on the surface. Cycling landscape is defined as a dummy variable where 0 indicate the gate is on a route that has no dedicated cycle lane and 1 indicates the gate is on a route that has dedicated cycle lanes.

#### 3.4.3. London Cycle Network

The third measure of safety and infrastructure is the designation of the London Cycle Network. The London Cycle Network (LCN) programme started in 1995 to designate and improve cycling routes in the city. The London Cycle Network can range from segregated cycle tracks, cycle lanes, shared paths with pedestrians, motor traffic speed reduction, road markings, and to the rectification of potholes [22]. The London Cycle Network variable for the cyclist movement model is specified as a dummy variable where 0 indicates the gate is not on a London Cycle Network and 1 indicates the gate is located on a London Cycle Network.

#### 3.4.4. Number of Vehicular Lanes

The fourth measure of safety and infrastructure is the number of vehicular lanes per segment. This dataset has been collected on site and verified on Google Streetview. The number of vehicular lane variable is specified as a discrete variable between 0 to 8 indicating the number of lanes on each street segment and gate location.

### 3.5. Character and Landuse

The presence of active land uses along a cycling route improves convenience in trip-chaining, the sense of security and the urban vitality of a route. It also increases the opportunities for economic transactions, as it is more likely that cyclist would to stop by on their way instead of diverting their route to consume. As a result measure of active land use is proposed for the cyclist movement model. This variable is specified as a dummy variable where 0 indicate the gate has no active/retail land use and 1 indicates the gate has active/retail land use. More research is needed in identifying more suitable instruments to measure the perceived character of urban spaces, such as vistas, green coverage, and quality of public spaces.

## 4. The Elephant and Castle Case Study

**Figure 4:**
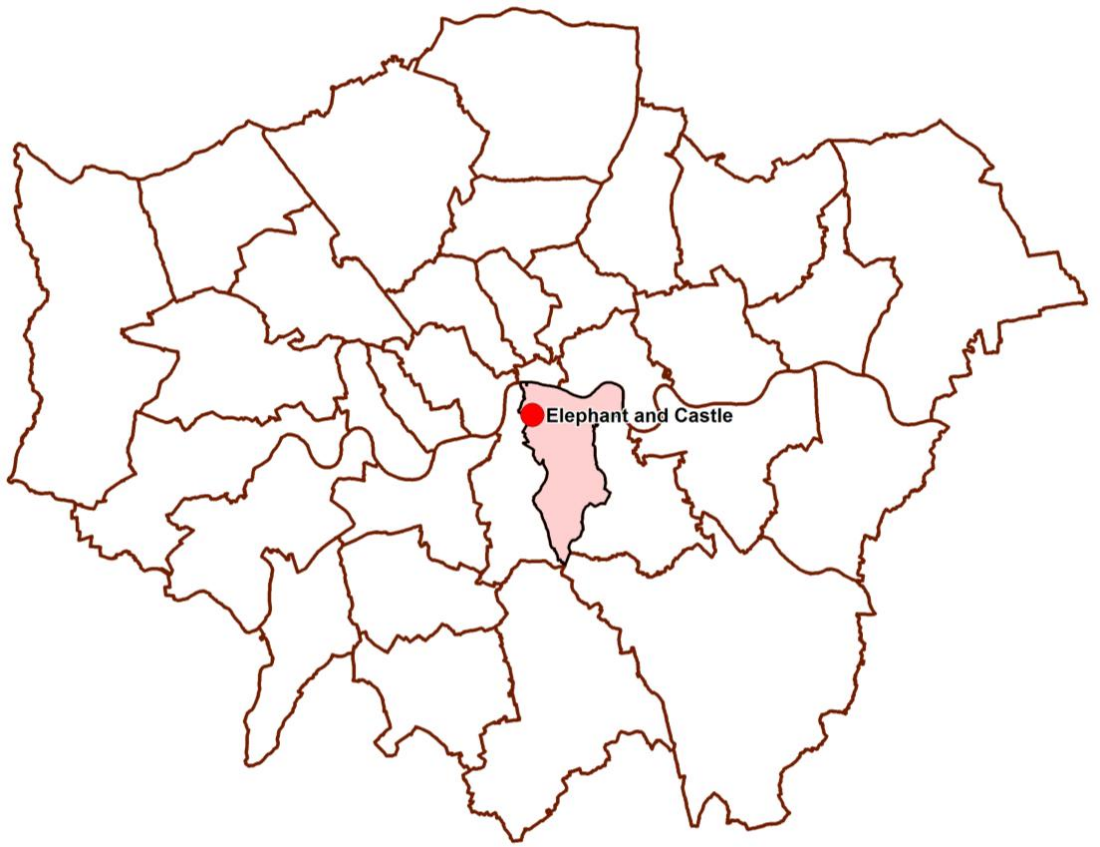
The figure describes Elephant and Castle, highlighted with a red dot in London Borough of Southwark.

This section analyses the Elephant and Castle case study in London, United Kingdom. Figure 4 illustrates the location of Elephant and Castle in London, highlighted with a red dot, in the London Borough of Southwark, highlighted with a pink polygon [23]. The first stage of the case study analysis is the cyclist movement analysis that describes the spatial configuration of the study area, its land use distribution and its cyclist movement pattern for both 2003 and 2012. The second stage of the case study is the construction of the cyclist movement model where cyclist movement is regressed against the variables identified in the research approach section.

Contains Ordnance Survey data © Crown copyright and database right 2014.

### 4.1. Background

**Figure 5:**
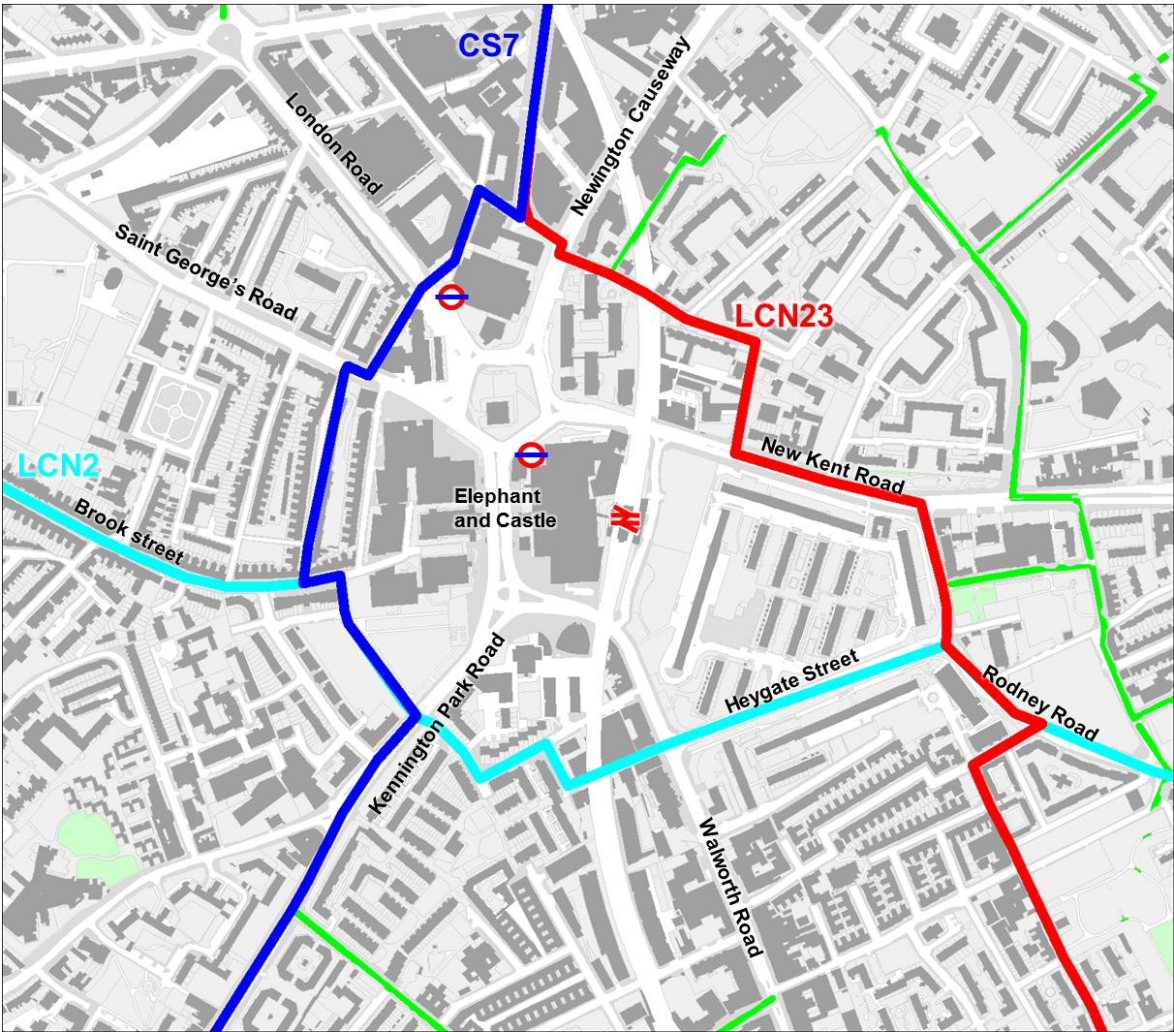
The figure describes the Elephant and Castle study area with the London Cycle Network; LCN23 in red, LCN02 in aqua, and London Cycle Superhighway CS7 in blue and green lanes in green.

Elephant and Castle is located in London Borough of Southwark. The study area is bordered by Kennington Road, Lambeth Road, Great Dover Street and New Kent Road. Figure 5 illustrates the study area for the case study and the existing cycling routes in the study area. The dark blue line represents the London Cycle Superhighway 07 (CS7) which covers the route from Colliers Wood to Southwark Bridge. The light blue line is the London Cycle Network Route 02 that runs from Brook Drive (Imperial War Museum) to Deptford. The red one is the London Cycle Network Route 23, from Southwark Bridge to Crystal Palace.

Contains Ordnance Survey data © Crown Copyright/database right 2014. An Ordnance Survey/EDINA supplied service.

### 4.2. Space Syntax Measures and Land Use Distribution of Study Area

**Figure 6:**
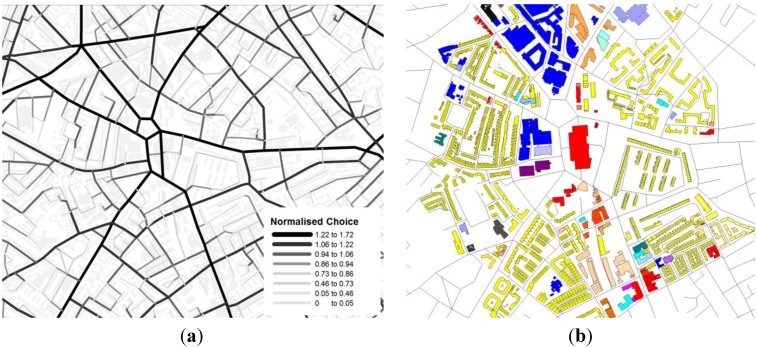
(**a**) Space syntax normalised angular choice of the case study area (**left**); (**b**) Land use distribution of the case study area (**right**).

The spatial network model for the case study uses the Ordnance Survey (OS) Mastermap Integrated Transport Network (ITN) dataset [24] which was then modified both manually and automatically using customized GIS geoprocessing tool at Space Syntax Limited into a road centre line segment model. The road centre line segment model yields similar results compare to a traditional axial map constructed in space syntax analysis [25]. Robustness in model representation needs to be validated for future research. Figure 6a shows the space syntax normalised choice map visualised using the colour spectrum: red for high accessibility through to orange, yellow, green and blue for areas with low accessibility. UCL Depthmap 10 was used to calculate the two spatial accessibility measures. [26] Looking at the spatial accessibility of the area, the radials converging at the Elephant and Castle-Walworth Road, New Kent Road, Elephant and Castle, St George’s Road, London Road, and Newington Causeway have the highest normalised space syntax angular choice values. Figure 6a,b shows similarities between the normalized angular choice of the study area and the land use distribution on the basemap created from the OS Mastermap Topography dataset [27]. In particular Elephant and Castle and Walworth Road have high normalized angular choice and active land use. 

Contains Ordnance Survey data © Crown Copyright/database right 2014. An Ordnance Survey/EDINA supplied service.

### 4.3. Cycling Movement Pattern

In order to understand the existing cycling movement patterns of the area, two sets of observational studies have been conducted in 2003 and 2012. In 2003, the observation was taken in over 50 locations in the area. Human observers recorded cyclist’s movement for five minutes each hour from 10:00 hours to 20:00 on Thursday, 20^th^ February 2003. In 2012, camera-based cyclist movement survey was carried out in 22 different locations throughout the day, from 07:00 to 19:00 on Tuesday, 21^st^ August 2012. A limitation of the study is differences from seasonal cyclist’s movement trends. 

**Figure 7:**
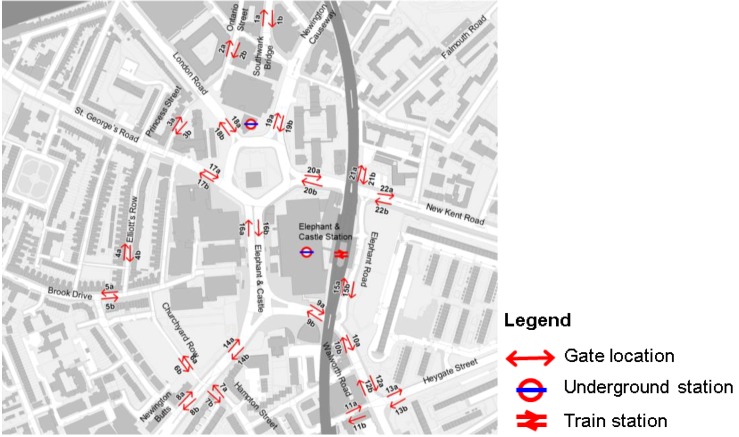
This figure highlights the gate locations for the cyclist movement survey.

For the survey 5 min counts were extracted at each location every half hour. Later on, the results were transformed into hourly movement rates for each hour and then an all day average cyclists per hour is calculated for the two samples. There are 21 locations where there are overlaps between the 2003 and 2012 observations. The small sampling of the panel study represents one of the challenges in gathering movement data across time. Figure 7 shows these gate locations.

**Figure 8:**
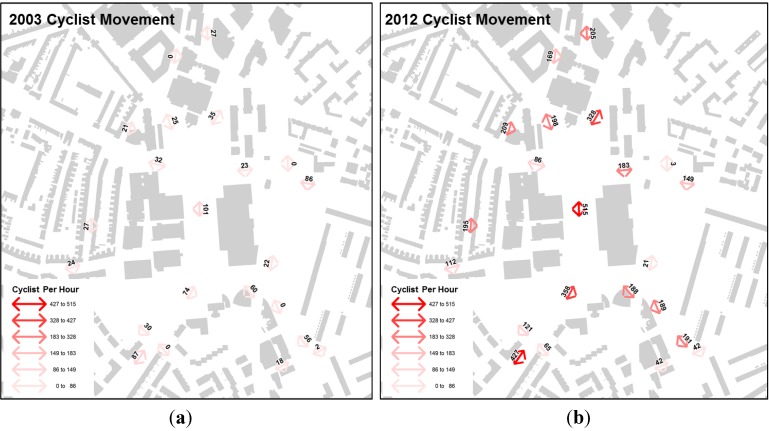
(**a**) Average all-day hourly cyclist movement pattern for the case study area in 2003 (**left)**; (**b**) Average all-day hourly cyclist movement pattern for the case study area in 2012 (**right**).

Figure 8 shows the recorded average all day cyclists per hour flow on a weekday for both 2003 on the left and 2012 on the right using the same ranges for visualisation. The all day average cyclists flows are visualised using the colour spectrum: red for high movement levels through to orange, yellow, green, and blue for areas with low movement level. Both of these data have been collected by Space Syntax Limited.

© Crown Copyright/database right 2014. An Ordnance Survey/EDINA supplied service.

Contains Ordnance Survey data © Crown Copyright/database right 2014. An Ordnance Survey/EDINA supplied service.

#### 4.3.1. Cycling Movement Pattern 2003

Figure 8a shows high levels of average all day hourly cyclist movement in 2003 on the primary routes in the network—Newington Butts, Walworth Road and New Kent Road—where the radials converge in Elephant and Castle. In contrast, cyclist movement rates are significantly lower in the secondary network, between the radials and especially in residential streets. This distribution suggest on aggregate cyclist’s preference for direct and highly accessible routes in the street network. The highest movement of 60+ cyclists per hour is observed along Elephant and Castle, Newington Butts, and New Kent Road, 40–60 cyclists per hours along St.George’s Road, London Road and Walworth Road and the rest of the routes below 40 cyclists per hour.

#### 4.3.2. Cycling Movement Pattern 2012

Figure 8b shows a significant overall increase in 2012 as compared to 2003 cyclist movement pattern. In contrast, cyclist movement distribution increased significantly on Elliot’s Row, which is part of London Cycle Superhighway CS7. During the morning period the dominant movement follows Newington Butts, Elephant and Castle and Newington Causeway heading north towards Southwark Bridge and London Bridge. An important secondary movement line was recorded along Elliott’s Row, Princess Street, Ontario Street, and Southwark Bridge, which overlaps with CS7. Medium levels of cyclist movement were recorded on the Northern Roundabout radials: London Road, St. George’s Road, and New Kent Road, as well as Walworth Road leading onto the Southern Roundabout.

#### 4.3.3. Comparing Cycling Movement Pattern between 2003 and 2012

**Figure 9:**
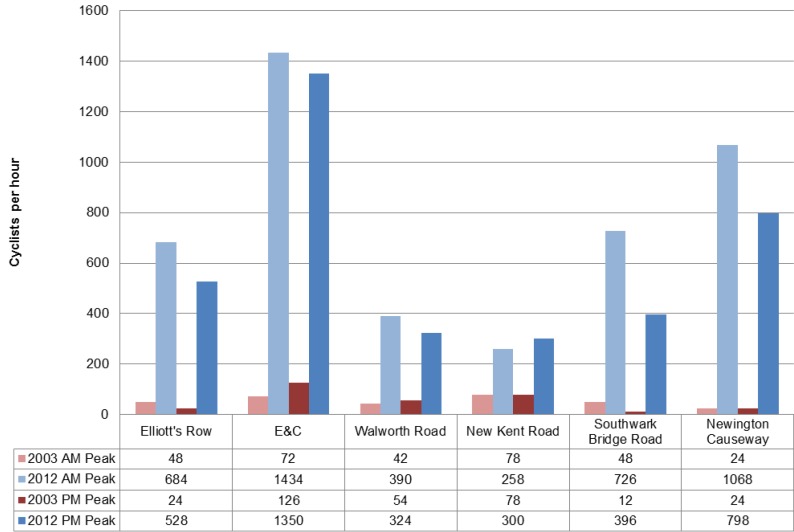
Bar Chart shows in red 2003 AM peak and PM peak cyclists movement and in blue 2012 AM peak and PM peak cyclists movement for six street segments in the study area.

Taking all into consideration, a comparison between 2003 and 2012’s cycling numbers shows a 600% average cycling activity increase in the area. Elliot’s Road, along a quiet but segregated section of CS7, recorded the largest increment. However, when taken as a whole, 68% of cyclists prefer the Elephant and Castle over Elliot’s Row along London Superhighway CS7 between the Northern and Southern roundabout. This result is in line with previous research findings where more cyclists are observed on major routes which offer direct connections than secondary quieter roads despite the introduction of dedicated cycling lanes along the cycling superhighway. Figure 9 shows a bar chart in red 2003 AM peak and PM peak movement and in blue 2012 AM peak and PM peak movement for six street segments in the study area. Street segments include Elliott’s Row, Elephant and Castle, Walworth Road, New Kent Road, Southwark Bridge Road and Newington Causeway.

**Figure 10:**
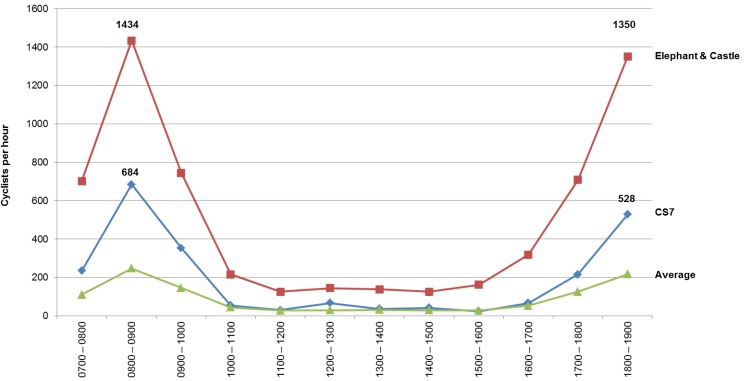
Cyclist movement distribution across the time comparison.

Figure 10 shows cycling movement distribution across time where the X axis shows the time period and Y axis shows the average cyclist per hour. The red line show the Elephant and Castle route, the blue line show Elliott’s Row on CS7, the green line shows average cyclist movement for all gates. Qualitatively, faster commuter cyclists were observed more on Elephant and Castle whilst slower non commuter cyclists were observed on CS7. The difference between the most accessible route over the safer designated CS7 route persist throughout the day.

**Figure 11:**
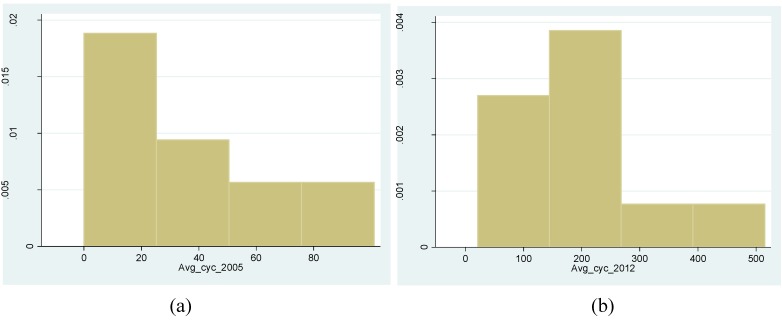
(**a**) Histogram depicting average all-day hourly cyclist movement distribution for the case study area in 2003 (**left)**; (**b**) Histogram depicting average all-day hourly cyclist movement distribution for the case study area in 2012 (**right**).

Table 2 summarises the 21 overlapped observations for the two years 2003 and 2012. The mean and standard deviation of the all-day cyclists’ hourly movements are 36/30 respectively in 2003 and 190/126 respectively in 2012. This shows the significant increase in cyclist movement for the same gates in the study area between the two time periods. Figure 11 shows the histogram for the two years 2003 and 2012 indicating the majority of the movement is on the minority of the segments.

**Table 2:** Cyclist movement distribution across the time comparison.

Variables	Obs	Mean	Std. Dev.	Min	Max
avg_cyc_2003	21	35.714	30.152	0	101
avg_cyc_2012	21	190.143	126.801	21	515

**Figure 12:**
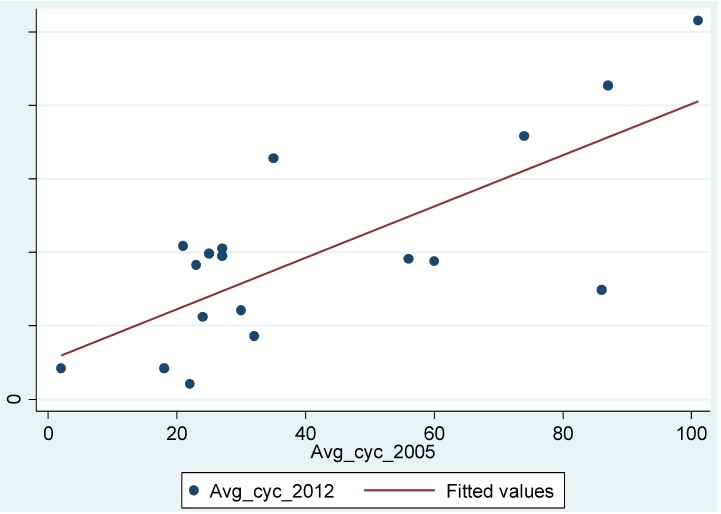
The figure highlights a scatterplot between 2003 and 2012 observed average cyclist movement.

Figure 12 shows the scatterplot and fitted line between observed cyclist movement in 2003 and 2012. Three overlapped gates with zero movement have been discarded in 2003 for the purpose of correlation. As a limitation of the study, the percentage is likely to be over-estimated due to seasonal differences. This does not reduce the validity in examining relative increases across the two time periods. However future studies are recommended to validate this relationship.

Table 3 reveals that the 55% in the variation of the cyclists’ movement in 2012 can be explained by its variation in 2003. This result suggests, the route hierarchy and movement distribution in 2012 is dependent on cyclicsts movement in 2003 despite changes in both modal shift and increase in cyclist infrastructure. The result suggests the relationship between spatial configuration and aggregated cyclist movement distribution is consistent across the two time periods. Routes with higher movement overtime are also routes that are least angular, faster for cyclists and requiring less cognitive information to traverse. Statistically, this preference for the same route between observed cyclists in the two time periods correspond to the skewed distribution of movement patterns where the majority of cyclists are on the minority of the segments. Further research is needed into examining this relationship.

**Table 3:** Regression results between cyclist movement in 2003 and cyclist movement in 2012.

	(1)OLS
VARIABLES	(Avg_Cyc_2012)
Avg_cyc_2003	3.491 ***
	(0.790)
Constant	52.86
	(39.49)
Observations	18
R-squared	0.550

Notes: Standard errors in parentheses; *** *p* < 0.01, ** *p* < 0.05, * *p* < 0.1.

## 5. Cyclist Movement Model

In order to validate the consistent relationship between accessibility and cyclist movement as well as to identify the key factors in influencing observed aggregated cyclists movement, the cyclists’ movement model is proposed. The cyclist movement model is a statistical model split into two stage. The first stage is to explore and examine the datasets through multivariate scatterplots between Log cyclists’ movement in 2003 and 2012 and space syntax accessibility measures as specified in Section 3.3. The second stage is model selection where a stepwise regression method is used to examined and select statistically significant cyclist movement model. The full cyclists movement model is then computed for the 2012 datasets.

### 5.1. Exploratory Data Analysis

**Figure 13:**
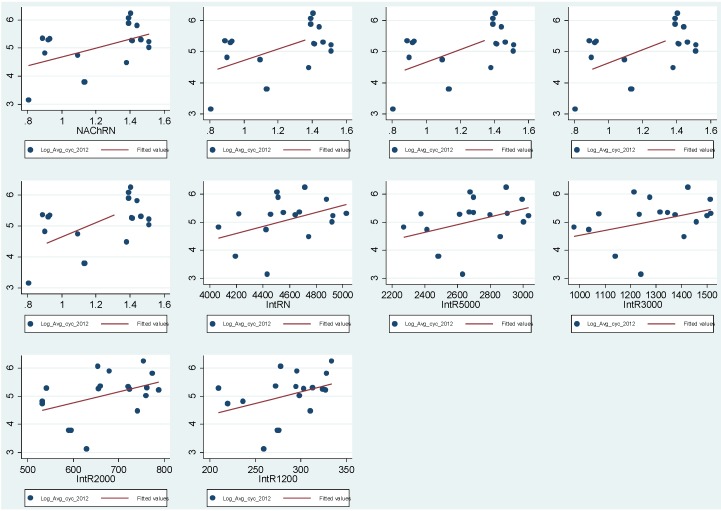
The figure highlights the Log-Log Scatterplots between Log cyclists’ movement 2012 and different radii of Space Syntax measures.

Figure 13 illustrates the log-log scatterplots between both Log cyclists movement in 2012 and Space Syntax normalized angular choice measures and space syntax normalized angular integration measures. The scatterplots shows a positive relationship between cyclists’ movement and space syntax measure in 2012.

**Figure 14:**
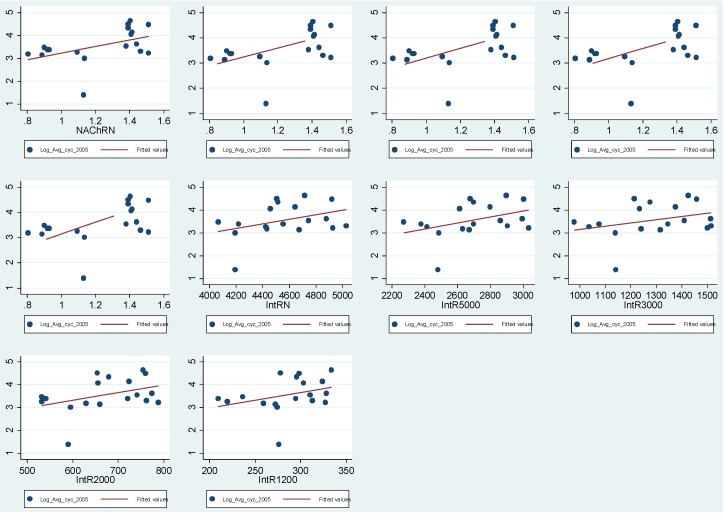
The figure highlights the Log-Log Scatterplots between Log cyclists’ movement 2003 and different radii of Space Syntax measures.

Figure 14 illustrates the log-log scatterplots between Log cyclists’ movement in 2012 and Space Syntax normalised angular choice measures and Space Syntax angular integration measures. The scatterplots shows a positive relationship between cyclists’ movement and space syntax measure in 2003.

**Table 4:** Pearson correlation coefficient between average cyclist movement in 2003 and 2012 across different accessibility measure.

Pearson Correlation Coefficient [R]	Cycling Movement 2003	Cycling Movement 2012
NAChR1200	32%	29%
NAChR2000	35%	34%
NAChR3000	37%	37%
NAChR5000	38%	39%
NAChRN	40%	46%
IntR1200	28%	41%
IntR2000	36%	45%
IntR3000	29%	42%
IntR5000	39%	42%
IntRN	40%	47%

### 5.2. Stepwise Regression Model Results

A stepwise regression model is conducted as part of the model selection process. The stepwise regression optimises model selection employing a backward stepwise strategy in discarding variables where the statistically significant probability threshold of rejecting the null hypothesis is 0.05. The variable in the multiple variable regression is discarded when the probability significance is greater than the 5% level (Prob ≥ 0.05). Table 5 illustrates the results from the stepwise regression variable selection process between Log cyclists’ movement and Normalised Choice Radius N plus each variables from Table 1 (6). The normalized angular choice radius N measure is used as a base variable due to its higher explanatory power in the multiple variable regression than the space syntax angular integration measure. The variable for the number of lanes had also been dropped due to high multi-collinearity between number of lanes (supply) and accessibility (demand).

*Log*(*Cyclists movement*) = ∑_i_*α*_i_*Accessibility* + ∑_k_*β*_i_*Safety* + ∑_k_γ_i_*Character* + *ε* (6)

**Table 5:** Stepwise regression variable selection process.

Stepwise Regression Variable Selection	*p-Value Threshold* *(If p* ≥ *0.05 remove variable)*
removing LCN	*p* = 0.9440 ≥ 0.0500
removing Active	*p* = 0.2538 ≥ 0.0500
removing Landscape	*p* = 0.1833 ≥ 0.0500

The model starts with all the variables in the backward stepwise regression model. The London Cycle Network variable was removed in the first stage of the stepwise regression model with the probability = 0.94 indicating the variable is insignificant. The presence of active land use was removed next with the probability = 0.25 indicating the variable is insignificant. The presence of cycling landscape was removed last with the probability = 0.18 indicating the variable is insignificant. The variable for the presence of active land use, London Cycle Network and cycling landscape were discarded from the model selection process. For robustness reasons, a forward stepwise strategy which is not reported in the paper yield the same results.

To further validate these results, four separate regression model are constructed for each of the discarded variables and reported. The method of ordinary least square (OLS) is used for the estimation of the normal linear quadratic regression model. The first regression model regress Log cyclists movement with Normalised Choice as the base plus the presence of London Cycle Superghighway (7), the second with the presence London cycle network (8), the third with the presence of cycling landscape (9), and the fourth with the presence of active land use (10)


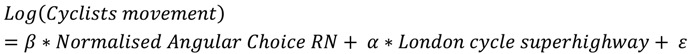
(7)


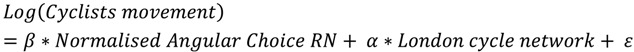
(8)


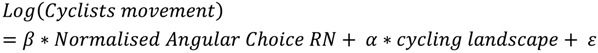
(9)


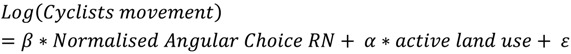
(10)

Table 6 is a statistical report for the four regression models yielding similar results as per the stepwise regression model. London Cycle Superhighway achieved a statistical significance at the 1% level whilst London cycle network and cycling landscape achieved a statistical significance at the 5% level. The active land use factor was not statistically significant. Model 1 with the London cycle superhighway achieved the highest goodness of fit with a R-square of 65.6%, model 2 with the London Cycle Network achieved a R-square of 38.8%, model 3 with London Cycling landscape achieved a R-square of 41.8% and model 4 with the presence of active land use achieved a R-square of 21.3%. This result confirms the previous stepwise regression method indicating the significance of the spatial configuration variable and london cycle superhighway variable.

**Table 6:** Cyclist movement model stepwise regression results.

Model	(2) OLS	(3) OLS	(4) OLS	(5) OLS
Variables	London Cycle Superhighway	London Cycle Network	Cycling Landscape	Active Land use
NAChRN	2.953 ***	0.954	2.478 ***	1.462 **
	(0.532)	(0.606)	(0.695)	(0.682)
LCS2	1.402 ***			
	(0.290)			
LCN2		−0.799 **		
		(0.351)		
Landscape2			0.919 **	
			(0.364)	
Active2				−0.0613
				(0.347)
Constant	1.042	4.052 ***	1.711 *	3.285 ***
	(0.704)	(0.779)	(0.929)	(0.800)
Observations	21	21	21	21
R-squared	0.656	0.388	0.418	0.213

Note: Standard errors in parentheses; *** *p* < 0.01, ** *p* < 0.05, * *p* < 0.1.

### 5.3. Cyclist Movement Model Regression Results

The selected cyclists movement model regressed Log cyclists movement against accessibility and cycle superhighway as defined below (11).


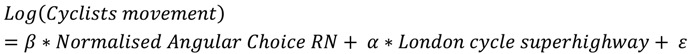
(11)

The method of ordinary least square (OLS) is used for the estimation of the normal linear quadratic regression model where the assumption of the normality of residuals and collinearity are checked. The regression results using OLS are presented in Table 7.

**Table 7:** Cyclist movement model regression results.

	(6)
VARIABLES	London Cycle Superhighway and NAChRN
NAChRN	2.953 ***
	(0.532)
LCS2	1.402 ***
	(0.290)
Constant	1.042
	(0.704)
Observations	21
R-squared	0.656

Note: Standard errors in parentheses; *** *p* < 0.01, ** *p* < 0.05, * *p* < 0.1.

The result suggests 65.6% of the variation in cyclists movement can be explained jointly by the combination of Normalised Choice Radius N and the presence of London Cycle Superhighway. Both variables are statistically significant at the 1% level. The higher coefficient estimates for Normalised choice indicate spatial configuration variable has statistically greater explanatory power on cyclist movement than the London Cycle Superhighway variable for the case study. These results confirm our earlier findings where despite the improvements in cycling landscape, the accessibility of the route is statistically more important in explaining aggregate cyclist movement. For robustness purposes, interactions between independent variables have been tested. Due to the small sample size these results are not conclusive and therefore not reported formally. Future research is needed in validating the results from the cyclists’ movement model in different geography and a greater sample size.

## 6. Conclusions

It is undeniable that cycling activity is increasing in our cities, and, as the London case study shows, sometimes independently from improvements to cycling infrastructure. This paper has outlined four findings. First, on aggregate higher level of cyclists are observed on the most direct and continuous routes over routes which have better cycling infrastructure but are less direct. The directness is measured between all pairs of origins and destinations. This relationship persists throughout the day in this case study. This result is in line with previous research where on aggregate higher cyclists movement are observed on major routes offering direct connections than less direct routes [15]. Second, higher cyclist movement were observed on routes with better cycling landscape provision as illustrated by the introduction of London Cycle Superhighway 07 along Elliot’s Row. This relationship is also in line with previous research where cyclists prefer to use safer routes with more cycling landscape infrastructure provision [3,4]. Third, 56% in the variation of the cyclists’ movement in 2012 can be explained by its distribution in 2003. The result suggests the relationship between the spatial configuration and aggregated cyclist movement is consistent across the two time periods. Statistically, this preference for the same route corresponds to the scaling distribution of movement in spatial networks where the majority of cyclist movement is on the minority of the segments. Fourth, 65% of the cyclist movement can be explained jointly by space syntax accessibility variable and London cycle superhighway variable for this case study. The result confirms previous research, the relationship between spatial configuration variable [15] and the provision of cycling landscape [3,4] with aggregated cyclist movement. The active land use variable was not statistically significant. To end, from a spatial cognition perspective this research enriches our understanding on how the external built environment as measured by the urban spatial configuration relates to aggregate cyclists movement and in identifying key potential factors in influencing cyclist wayfinding. Further research is needed into validating the results and examining this relationship at an individual basis on cycling route choice. From a design perspective, the evidence in the research suggests to optimise the efficiency of cycling infrastructure in cities, improving cycling infrastructure in more spatially accessible location is recommended and prioritised.
